# Development and application of an indirect ELISA for detecting equine IgG antibodies against Getah virus with recombinant E2 domain protein

**DOI:** 10.3389/fmicb.2022.1029444

**Published:** 2022-11-10

**Authors:** Xiangshu Qiu, Xinyu Cao, Ning Shi, He Zhang, Xiangyu Zhu, Yan Gao, Zhanhai Mai, Ningyi Jin, Huijun Lu

**Affiliations:** ^1^College of Animal Sciences, Institute of Preventive Veterinary Medicine, Zhejiang University, Hangzhou Zhejiang, China; ^2^Changchun Veterinary Research Institute, Chinese Academy of Agricultural Sciences, Changchun, Jilin, China; ^3^College of Veterinary Medicine, Northwest A&F University, Yangling, Shaanxi, China; ^4^Key Laboratory of Zoonoses Research, College of Veterinary Medicine, Ministry of Education, Institute of Zoonosis, Jilin University, Changchun, China; ^5^College of Veterinary Medicine, Xinjiang Agricultural University, Ürümqi, China

**Keywords:** Getah virus, ELISA, E2 domain, seroepidemiological investigation, Xinjiang

## Abstract

Getah virus (GETV) disease is a mosquito-borne infectious disease that causes fever, aseptic meningitis, and abortion in a variety of animals. Currently, the epidemic trend of GETV disease increases seriously worldwide, especially in China, posing a potential threat to animal safety and public health. However, there are few reports about the epidemiological investigation of GETV disease in China as well as a lack of commercial diagnostic kit for GETV antibody. Therefore, the establishment of a rapid, sensitive and suitable GETV antibody detection method for large-scale samples is an urgent request to fully understand the prevalence of GETV disease. Here, a recombinant plasmid pET22b-GETV-E2d that contained the domain of GETV-E2 (E2d) fused to His-tag was constructed to express recombinant protein E2d (rE2d) in *Escherichia coli*. The rE2d was mainly expressed in inclusion bodies. And it was purified successfully by nickel affinity column so that it could be used to develop an indirect ELISA (rE2d-ELISA). After optimizing reaction conditions of rE2d-ELISA, the cut-off value was determined as 0.396 with 100 equine sera tested by virus neutralization test (VNT). Furthermore, rE2d-ELISA method showed the positive rate of IgG antibodies against GETV was 54.3% based on testing 646 clinical serum samples obtained in Xinjiang whereas the overall coincidence rate between rE2d-ELISA and VNT was 94.0%, with 98.2% sensitivity and 92.6% specificity. The findings suggest that the developed IgG ELISA employing recombinant E2d promises was an efficient and low-cost type of antibody detection method for horse, which will benefit for prevention of GETV outbreaks in horses.

## Introduction

Getah virus (GETV) disease is caused by GETV infection and lead to nonspecific fever, aseptic meningitis, and abortion in a variety of animals. Since the first isolation of GETV strain MM2021 from the *Culex gelidus* in Malaysia in 1955 (Morita and Igarashi, 1984), it has been reported in China, Japan, Korea, Mongolia, Russia, Australia, India, and other Pan-pacific countries. Importantly, GETV disease is mainly transmitted by arthropods such as mosquitoes, which are abundant, diverse and widely distributed in tropical and subtropical regions, and easily exacerbate the transmission of GETV.

Getah virus is classified as a single-stranded, positive-sense RNA virus belonging to the genus *Alphavirus* of the family *Togaviridae*. Notably, the structural protein encoded by the E gene not only constitutes the surface protrusion of the viral particle, but is the critical protein for the adsorption of GETV virus to target cells and the induction of anti-infection immunity in the host (Voss et al., 2010). Based on the evolutionary analysis of the nucleotide sequence of the E2 gene or whole genome, GETV strains can be divided into four groups I–IV, and most of the strains prevalent in the world after 1964 belong to group III (Li et al., 2017; Shi et al., 2021). As a re-emerging mosquito-borne disease, GETV disease is an increasingly prominent epidemic problem in China. Related reports showed that before 2006, GETV was found in only six provinces in China (Zhai et al., 2008), but by 2018 the number of provinces affected by GETV increased dramatically to 15 (Lu et al., 2020). Besides, Horses infected with GETV show symptoms such as fever, hind limb edema, swollen submandibular lymph node and urticaria in multiple areas of the body. Seroepidemiological studies of GETV disease showed that the positivity rate of GETV antibody in horses was 17% in India (Brown and Timoney, 1998), 39% in Korea (Heo et al., 1987), and 24% in Hong Kong (Shortridge et al., 1994). In Japan, the infection rate in horses increased dramatically from 7.5 to 93% after GETV epidemic and that of wild boar was 47.8% (Imagawa et al., 1981). In China, Li detected antibodies to GETV in equine serum samples collected from Hainan Province in 1979, and the positivity rate was 25% (Li et al., 1992). Lu identified the GETV strain in 2018 from the blood of a febrile horse at a racecourse in Guangdong Province (Lu et al., 2019). The prevalence and danger of GETV in equine herds is becoming increasingly severe, but the available studies on GETV infection in equine populations in China are still less reported, especially in Xinjiang Uygur Autonomous Region where has the largest number of horses in China and borders eight countries such as India, Mongolia and Russia.

Although isolation and identification of virus is the most intuitive method to confirm GETV infection (Fukunaga et al., 2000), it is laborious and susceptible to failure. Molecular biological techniques include RT-PCR, RT-qPCR, loop-mediated isothermal amplification and random amplified polymorphic DNA (Hu et al., 2012; Liu et al., 2019). Serological methods include enzyme linked immunosorbent assay (ELISA), Virus neutralization test (VNT), immunofluorescence method, etc. (Wekesa et al., 2001; Bryant et al., 2005). Among them, ELSIA is the most widely used immunological technique because it is rapid, sensitive, economical and especially suitable for the detection of large quantities of samples. For instance, some ELISA methods for detecting antibodies against GETV were established using unique peptides, full length of E2 protein (Bannai et al., 2019, 2020) as diagnostic antigens. Nevertheless, it should be noted that the GETV-positive sera used in Bannai’ELISA assays were prepared by artificially infecting laboratory horses, which limited the wide utilization of these methods in other conventional laboratories because of the high cost of experimental horses and strict standard of laboratory biosafety condition for animal infection. The E2 domain (E2d), located at amino acids (aa) 12–335 of E2 protein (422 aa), has high conservation among different GETV strains (shown as in Supplementary Table 1) and mainly consists of three main structural domains (A, B, and C). Crucially, E2d possesses unique antigenic epitopes for inducing the production of neutralizing antibodies. According to this, antigenic epitopes of E2d have been used as target sites for the development of serological diagnostic tests for Chikungunya virus (Cho et al., 2008; Morey et al., 2010; Verma et al., 2014), which also belongs to the alphavirus with GETV. Therefore, E2d, with high conservation and perfect immunogenicity, has the potential to be used as coating antigens to establish an ELISA assay for GETV antibody.

Above, the objective of this study is by preparing recombinant E2d protein using prokaryotic expression system to develop an effective and rapid ELISA method for detecting equine GETV antibodies, providing a powerful tool for the seroepidemiological investigation of GETV disease in horse in Xinjiang, China.

## Materials and methods

### Virus, cells and serum samples

The GETV strain SD17/09 (GenBank No. MH106780.1) was saved in our laboratory (Shi et al., 2019). Vero cells (ATCC number: CCL-81) were obtained from ATCC (United States) and cultured in DMEM containing 10% fetal bovine serum (FBS; Solarbio, China). All mediums were supplemented with 100 U/ml penicillin, and 100 U/ml streptomycin. Positive serums of equine infectious anemia virus (EIAV), equine influenza virus (EIV) and salmonella were purchased from Guosheng Biological Company, China. The ascitic fluids from mice immunized with ross river virus (RRV) were purchased from ATCC (United States). Positive serums of tetanus and GETV (neutralizing antibody titer is 1:256) with negative GETV serum were saved in our laboratory. 646 equine serum samples were collected from horses not inoculated with GETV vaccine from June to September 2021, Xinjiang (Changji, Ili, Aksu and Kashi), China.

### Construction of recombinant plasmid

**Figure 1:**
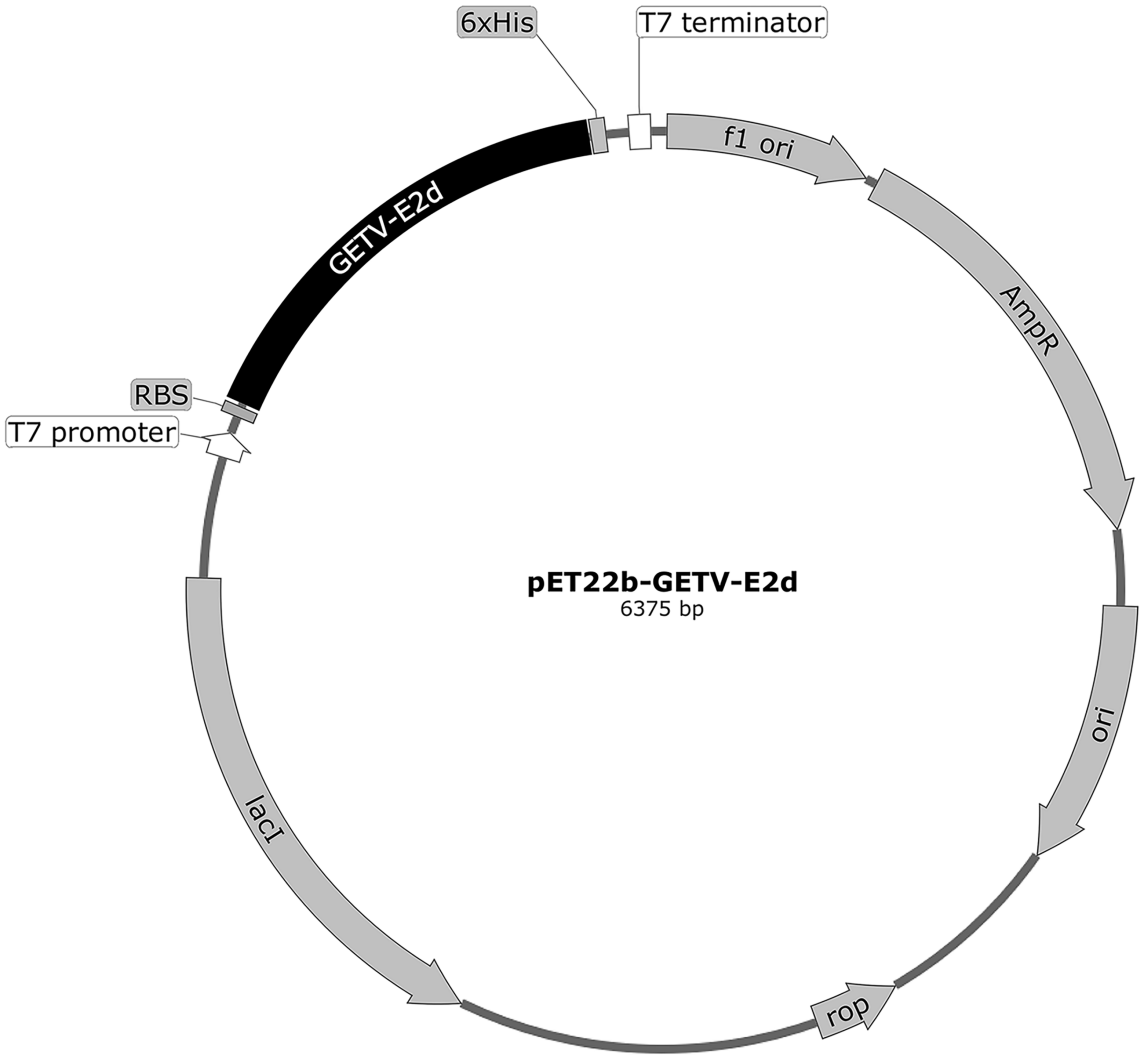
The circle map of GETV-E2d-His fusion expression plasmid. The domain of E2 (E2d) locating 34–1,038 nt of E2 (1,266 bp) was 3′ terminally fused with a 6 × His sequence and then cloned into the *Nde* I–*Xho* I restriction sites of the prokaryotic expression vector pET-22b (+) to yield the recombinant plasmid pET22b-GETV-E2d.

To show the conservation of E2d among different GETV, MEGA X and DNASTAR were used to align and analyze using Clustal W method. All published E2d sequences of GETV were retrieved from the National Center for Biotechnology Information (NCBI; May 2022). To express the recombinant protein efficiently, recombinant plasmid containing E2d, 34–1,038 nt of E2 gene (1,266 bp), was constructed using the prokaryotic expression vector pET-22b (+) with a His tag applied to purify protein (Figure 1). The gene sequence of E2d was amplified from the genomic RNA of GETV strain SD17/09 by RT-PCR. The primers are as follows: forward primer, 5′-CGCATATGACGAAACCGTACCTAGCGTA-3′ and reverse primer, 5′-GCCTCGAGTTTGCCTTCAGTTGTCAGCT-3′. Underlined portions represent *Nde* I and *Xho* I restriction sites, respectively. The amplification product was 1,017 bp in length confirmed by agarose gel electrophoresis and sequencing followed by cloned into the *Nde* I–*Xho* I restriction sites of the prokaryotic expression vector pET-22b (+) and named pET22b-GETV-E2d.

### Expression and purification of recombinant protein

The recombinant GETV E2d protein was expressed in *E. coli* as previously described (Bannai et al., 2019). Protein expression was induced at 37°C for 12 h with isopropyl β-d-1-thiogalactopyranoside (IPTG) at 1.0 mM final concentration. The recombinant protein was extracted and purified according to the instruction of His-Tagged Protein Purification Kit (Cwbio, China) and then was re-folding by dialysis in a gradient of 6, 4, 2, 1, and 0 M urea buffer solution (20 mM Tris–HCl, 500 mM NaCl) at 4°C. The renatured protein used for ELISA development was identified by SDS-PAGE and Western blot, and its concentration was determined using a BCA Protein Assay Kit (Beyotime, China). 1:1,000 diluted Mouse Anti-His-Tag mAb (Beyotime, China) and 1:2000 diluted HRP-labeled Goat Anti-Mouse IgG (H + L; Beyotime, China) were used as antibodies in Western blot.

### rE2d based indirect ELISA (rE2d-ELISA) development

Common indirect ELISA detecting serum antibodies was established as described previously (Lin et al., 2018). Briefly, 100 μl/well of rE2d which was gradually diluted from 32 to 1 μg/ml in 50 mM bicarbonate buffer (pH 9.6) was coated on 96 well ELISA plates (Costar, United States) and incubated overnight at 4°C. After washing, the plates were blocked with 300 μl/well different blocking buffer including bovine serum albumin (BSA), skim milk (SM) or fetal bovine serum (FBS) followed by incubated for 2 h at 37°C. Then 100 μl/well standard equine sera diluted from 1:20 to 1:200 were added and incubated for 1 h at 37°C. Next, plates were reacted with 100 μl/well secondary antibody (Rabbit Anti-Horse IgG H&L, Bioss, China) at dilutions ranging from 1:2,000 to 1:5,000 for 1 h at 37°C. In order to visualized the peroxidase reaction, 100 μl 3,3′,5,5′-tetramethylbenzidine (TMB; Tiangen, China) as the substrate of HRP was add to each well for 15 min at 25°C in complete darkness and stopped by adding 100 μl of 2M H_2_SO_4_ (Solarbio, China). The plate was washed by 200 μl 1 × PBST (1 × PBS containing 0.1% Tween-20)/well/each time for three times before blocking buffer, serum, antibody or substrate was added. In the end, using microplate reader Spark-10 M (Tecan, Switzerland), each well’s OD_450_ value was measured and immediately recorded. The reaction conditions were considered optimal when the OD_450_ ratio between the positive and negative serum (P/N value) reached the highest and OD_450_ value of positive serum closed to 1.0 whereas that of negative serum was as low as possible.

### Determination of the cut-off value of rE2d-ELISA

Based on the detection results of 100 clinical sera (50 seronegative samples and 50 seropositive samples verified by VNT) using rE2d-ELISA methods, ROC curves and area under the curve were plotted, and ELISA sensitivity (Se) and specificity (Sp) were calculated. When the Youden index (Youden = Se + Sp–1) reached the biggest, a cut-off value for ELISA was determined, indicating that sample OD_450_ equal to or greater than the cut-off value was judged to be positive.

### Sensitivity, specificity, repeatability of rE2d-ELISA

To test the sensitivity of rE2d-ELISA, two folds serum dilutions ranging from 1:400 to 1:3,200 were tested. Five positive serums against EIV, EIAV, RRV, tetanus and salmonella, respectively, were used to verify the specificity of the method. To evaluate the intra-assay repeatability and inter-assay reproducibility, GETV positive serum were tested in five replicates in a same ELISA plate and five independent ELISA plates at different time, respectively.

### Detecting clinical equine sera using rE2d-ELISA and VNT

To further understand the prevalence of GETV disease in horses in Xinjiang, 646 clinical equine sera collected from Changji (181), Ili (175), Aksu (128) and Kashi (162) were detected for the presence of GETV antibodies using rE2d-ELISA developed in this study and VNT described as a previous report (Bannai et al., 2015) separately to analyze positive rate of antibody and the coincidence rate with consistency. Positive serum was defined if its neutralizing antibody titer was ≥ 1:4 in VNT. The geometric mean titer (GMT) of neutralizing antibody was calculated using titers of all positive equine sera verified by VNT.

### Statistical analysis

Coefficient of variation (CV), receiver operating characteristic (ROC) curve, and the correlation between rE2d-ELISA and VNT were performed using Excel and GraphPad Prism software. Coincidence rate = (true positive sample number + true negative sample number)/number of total samples. Chi-squared test was used to compare the difference of positive rate in different areas. Kappa value was determined by SPSS26.0.

## Results

### Expression and purification of GETV-E2d protein

**Figure 2:**
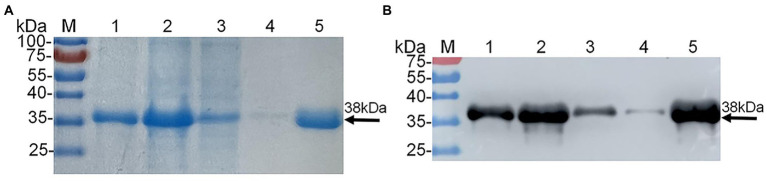
Expression and purification of recombinant protein E2d (rE2d) expressed in *Escherichia coli*. **(A)** SDS-PAGE. **(B)** Western-blot. M, protein marker; lane 1, rE2d supernatant; lane 2, rE2d inclusion bodies; lane 3, flow-through solution; line 4, washing solution; line 5, eluent.

As shown in Supplementary Table 1, homogeneous analysis demonstrated that the E2d of SD17/09 strain showed nucleotide conservation of 96.8–99.9% and amino acid conservation of 97.0–100% with other 70 GETV strains. Then recombinant plasmid pET22b-GETV-E2d was constructed according to Figure 1. Protein expression in positive colonies was induced using 1 mM IPTG, followed by incubation at 37°C for 12 h. After purification and dialysis, samples of rE2d were then analyzed by SDS-PAGE and Western blot. We verified that recombinant protein rE2d, approximately 38 kDa, was mainly expressed in insoluble inclusion bodies and could be purified by nickel affinity column successfully (Figure 2). The concentration of renatured protein was about 0.4 g/l.

### Establishment of rE2d-ELISA for detecting GETV antibodies

**Figure 3:**
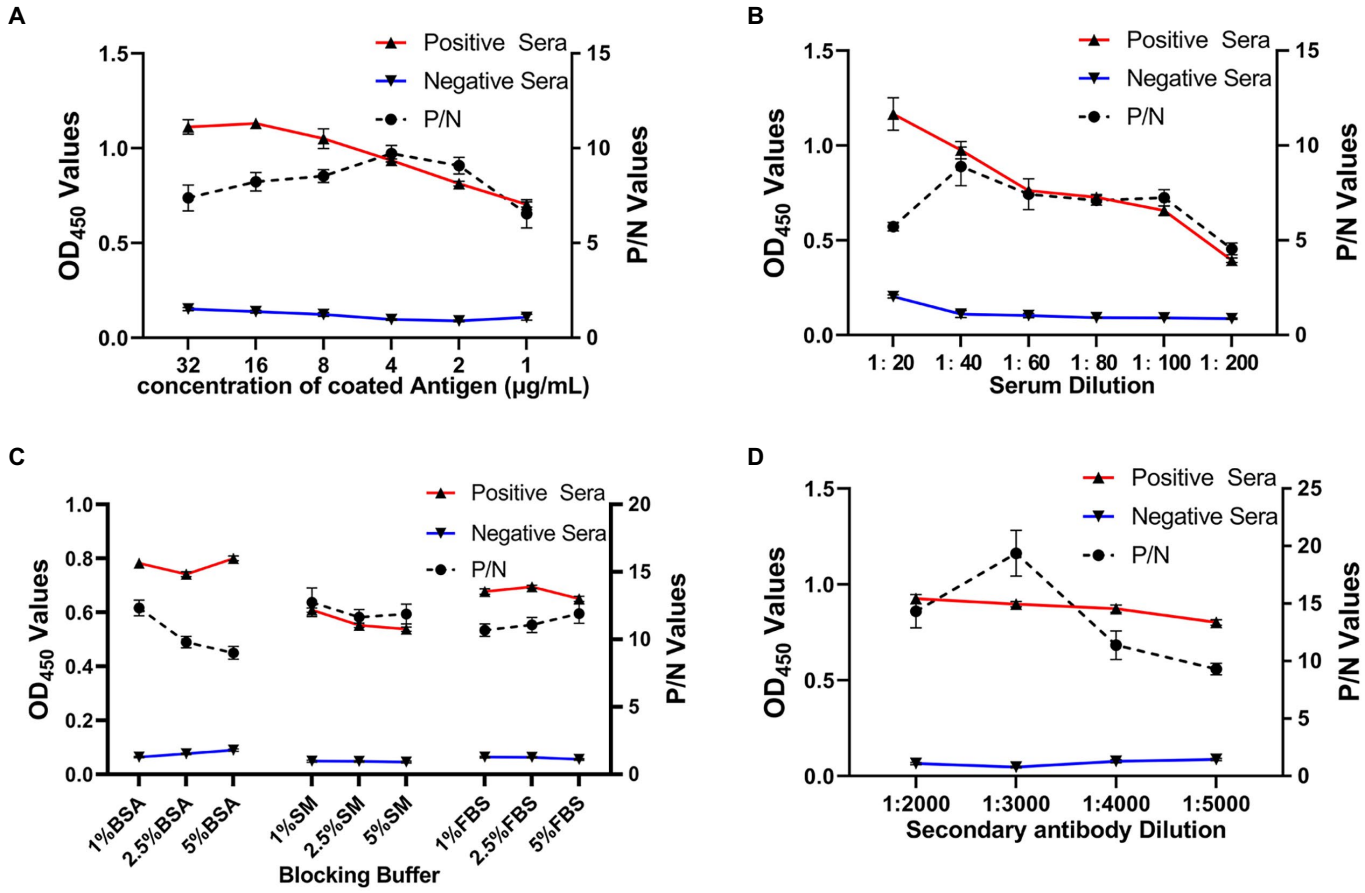
Optimization of rE2d-ELISA working conditions. Optimization of concentration of coating antigen **(A)**, serum sample dilution **(B)**, blocking buffer **(C)** and secondary antibody (HRP-labeled rabbit anti-horse IgG) dilution **(D)**. The reaction conditions were considered optimal when the OD_450_ ratio between the positive and negative serum (P/N value) reached the highest and OD_450_ value of positive serum closed to 1.0 whereas that of negative serum was as low as possible.

As shown in Figure 3, using a checkerboard titration method, the optimal concentration of antigen rE2d was found to be 4 μg/ml whereas the optimal dilution of serum and secondary antibody was 1:40 and 1:3,000, respectively. In addition, 1% BSA was the best blocking buffer with incubation condition being 37°C, 2 h. Furthermore, the reaction conditions of rE2d-ELISA were optimal when the reaction time of serum, secondary antibody and substrate was 90, 60, and 20 min, respectively, (data not shown).

### Determination of the cut-off value

**Figure 4:**
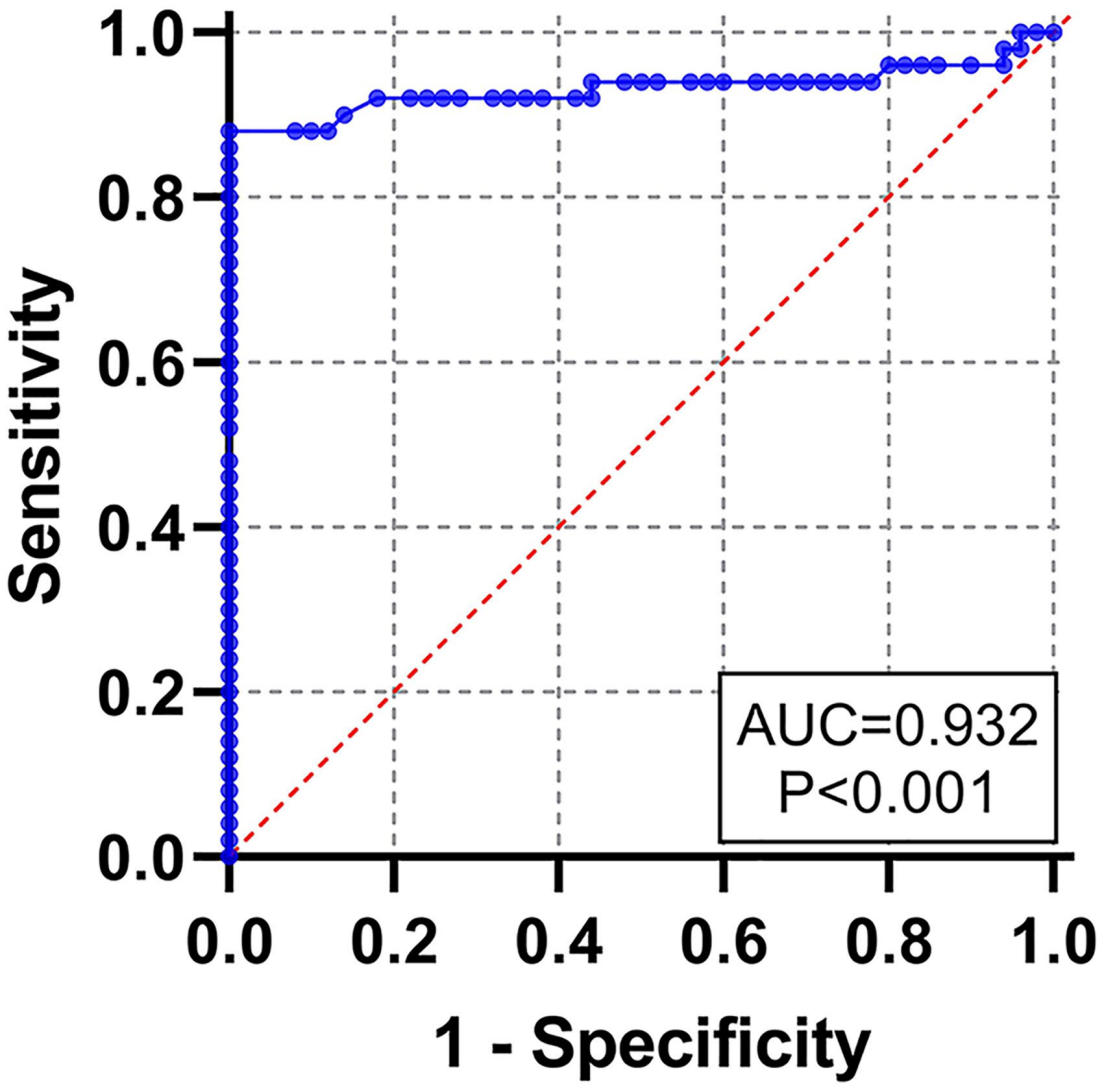
Receiver operating characteristic (ROC) curve for the rE2d-ELISA, based on the results obtained by the rE2d-ELISA and VNT with 100 serum samples.

To determine the cut-off value of rE2d-ELISA, 50 seronegative samples and 50 seropositive samples verified by VNT were tested using rE2d-ELISA method. An ROC curve analysis (shown as Figure 4) showed that the AUC value was 0.932, and the highest Youden index was 0.720 calculated by sensitivity (0.82) and specificity (0.90). Based on the biggest Youden index, the cut-off value was set at 0.396, indicating that the sample OD_450_ value ≥ 0.396 was considered to be GETV seropositive.

### Specificity, sensitivity, and repeatability of rE2d-ELISA

**Figure 5:**
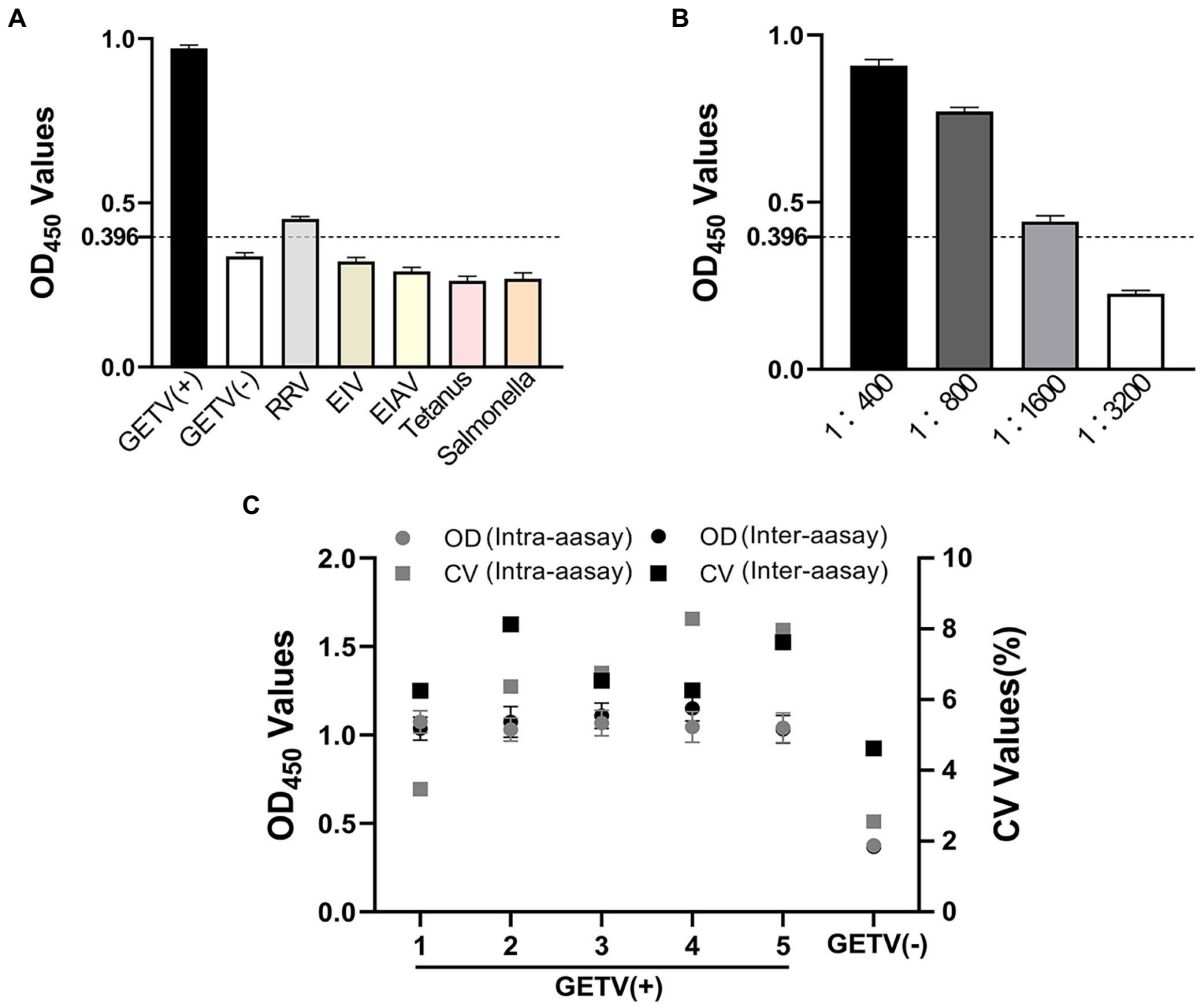
Sensitivity, specificity and repeatability of rE2d-ELISA. **(A)** Equine positive serums against Ross River Virus (RRV), equine influenza virus (EIV) H3N8, equine infectious anemia virus (EIAV), tetanus and salmonella were tested using the rE2d-ELISA and the mean OD_450_ value was calculated to determine whether the sample was positive. **(B)** Four dilutions of GETV positive serum were used to test the sensitivity of rE2d-ELISA. **(C)** Six control serum samples (five GETV positive sera and one GETV negative serum) were tested using rE2d-ELISA and OD_450_ values of serum samples were used to calculate CV values to determine the intra- and inter-assay reproducibility. The dashed line indicates the cut-off value (0.396) of rE2d-ELISA.

To assess the possible cross-reactivities of the rE2d protein with antibodies against common equine pathogeny, five kinds of positive serums including RRV, EIV, EIAV, tetanus and salmonella were tested in the rE2d-ELISA. The results showed the rE2 protein reacted weakly with positive sera against RRV (0.451) while such reaction was not observed in other positive sera, which were less than cut-off value(0.396) with 0.322, 0.291, 0.263 and 0.269 respectively, as shown Figure 5A. Sensitivity analysis proved that until the titer of the standard GETV positive serum was up to 1:1600, it still reacted obviously, which indicated the method had good sensitivity (Figure 5B). In the repeatability experiment, five GETV positive sera were used to determine the intra-assay and inter-assay CV of the rE2d-ELISA, which was 3.5–8.3% and 6.3–8.1%, respectively, as shown in Figure 5C.

### Detecting clinical equine sera using rE2d-ELISA

**Figure 6:**
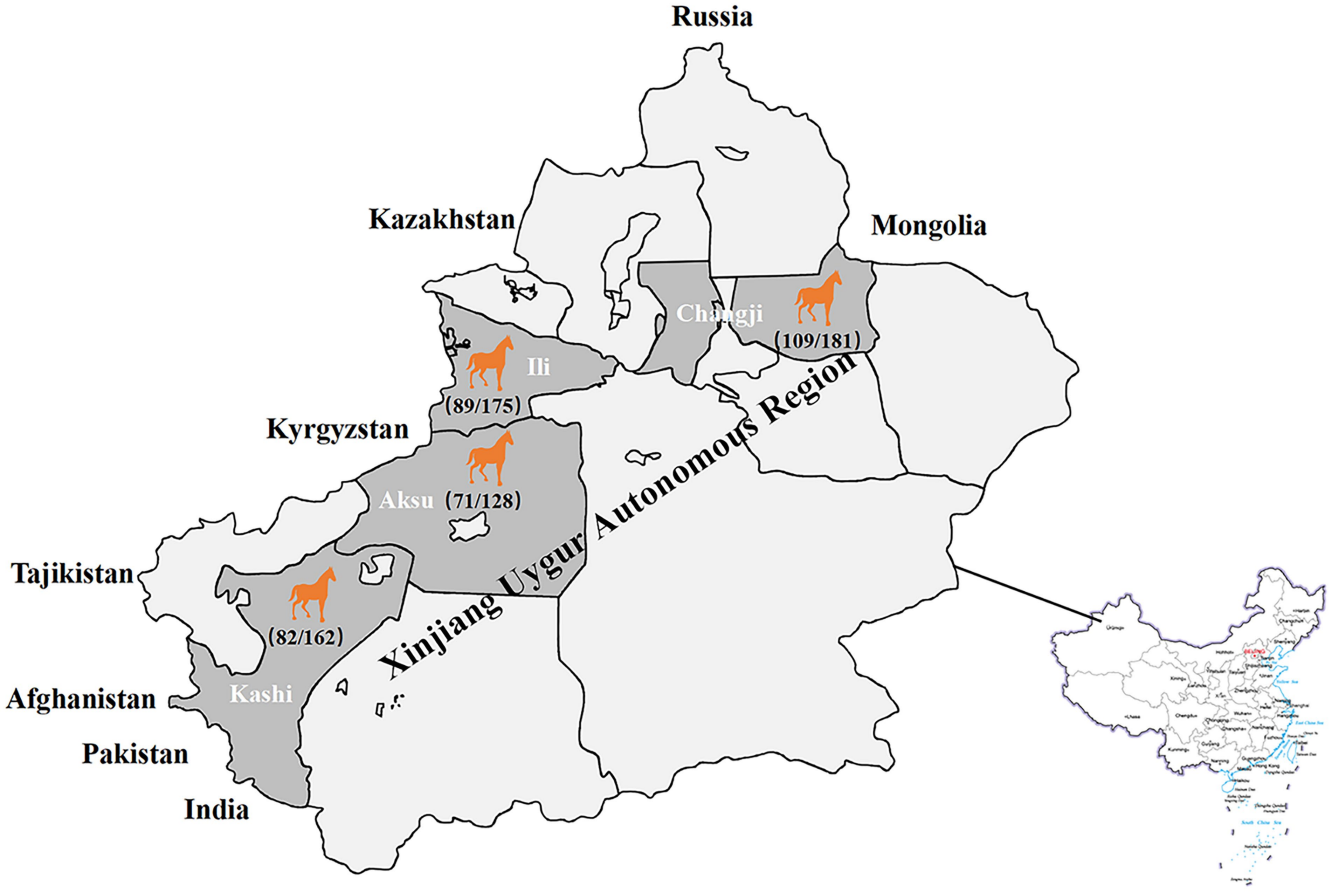
Serological surveillance of equine GETV disease in Xinjiang, China in 2021. The total positive rate of IgG antibodies against GETV in Xinjiang was 54.3% (351/646), and the positive rate in Changji, Ili, Aksu and Kashi was 60.2% (109/181), 50.9% (89/175), 55.5% (71/128) and 50.6% (82/162) respectively.

Seroepidemiological investigation showed that the total positive rate of IgG antibodies against GETV in Xinjiang was 54.3% (351/646; Table 1), and the positive rate in Changji, Ili, Aksu and Kashi was 60.2% (109/181), 50.9% (89/175), 55.5% (71/128) and 50.6% (82/162) respectively (Figure 6).

**Table 1:** Results of enzyme-linked immunosorbent assay based on recombinant protein GETV E2d (rE2d-ELISA) and virus neutralization test (VNT) applied to clinical equine serum samples.

		VNT		
		Positive	Negative	Total
rE2d-ELISA	Positive	328	23	351
	Negative	6	289	295
	Total	334	312	646
	Agreement	98.2% (328/334)	92.6% (289/312)	95.5% (617/646)

### Comparison of rE2d-ELISA with VNT

rE2d-ELISA method showed the total positive rate was 54.3% while that of neutralizing antibody determined by VNT was 51.7% (Table 1). Noted that the GMT value of neutralizing antibody in all 334 positive sera determined by VNT was calculated to be 1:135.8. Furthermore, the established rE2d-ELISA with 98.2% sensitivity and 92.6% specificity, had a high total coincidence rate (95.5%) and a decent consistency (Kappa value 0.879) with VNT (Table 1).

## Discussion

With its wide geographic distribution and strong host adaptation as shown in one of our previous reports (Shi et al., 2021), GETV disease has been seriously jeopardizing the healthy development of the horse and pig industries in China and even the world, causing large losses to the livestock industry and posing an increasing potential threat to public health security. Up to now, GETV disease outbreaks in equine herds six times worldwide, among which, five times occurred in Japan and one time in India. Even so, there is still no efficient commercial diagnostic kit for GETV antibodies, leading to serious inconvenience to both carry out epidemiological investigation of GETV disease and further strengthen the prevention and control of GETV disease. Taking ELISA’s advantages of sensitivity and suitability for the detection of large quantities of samples, some studies have reported that ELISA methods for detecting GETV antibody derived from pigs and horses could be established successfully using purified GETV virion as antigens (Lee et al., 2016). However, this method of preparing antigens utilizing virion may face the bio-safety risk of virus dispersal. Comparatively, it would be safer and more economical to use a prokaryotic or eukaryotic expression system expressing a recombinant protein with strong antigenicity to produce antigens. For example, based on a part of E2 protein, Li developed an ELISA method for testing antibodies against porcine GETV whose positive rate was low probably due to either the degradation of antibodies or the target gene did not contain the E2 domain (Li et al., 2012). Bannai selected full-length E2 and a peptide of E2 (151–170 aa) which was composed of beta-ribbon connector and domain B to established ELISA method, respectively, (Bannai et al., 2019, 2020). However, the epitopes of neutralizing antibodies in E2 protein were mainly concentrated in domains, and his study suggested that 46–65 aa and 196–245 aa located in the E2d also had good immunogenicity. In addition, the high cost of laboratory animals and the strict standard of laboratory biosafety condition for animal infection limit the use of these two methods in other conventional laboratories. In this study, compared with the full length of E2 (1–1,266 nt), we truncated the sequences at both ends of E2d that did not contain neutralizing antibody epitopes, and selected the whole E2 domain (34–1,038 nt) as the target protein, so that the epitopes were more concentrated and the antibodies produced by GETV infection could be detected to a greater extent. Moreover, the shorter sequences were more conducive to plasmid construction and protein expression. By the way, it was convenient to screen and obtain GETV-negative and GETV-positive sera from clinical serum samples by using VNT. Importantly, the overall agreement (95.5%) and Kappa value (0.879) of rE2d-ELSIA established in this study were higher than those of the above two methods (90.9%, 0.792 and 94.5%, 0.865), indicating that the detection results of E2d-ELSIA were closer to those of VNT.

The recombinant plasmid pET22b-GETV-E2d was successfully constructed in this study (Figure 1), and the recombinant protein rE2d was expressed mainly in the form of inclusion bodies with an expected size (38 kDa) identified by SDS-PAGE and Western-blot (Figure 2). Some researchers cloned the full-length GETV-E2 gene into the expression vector pET-47b and the recombinant protein mainly expressed as inclusion body (Bannai et al., 2019), which was basically consistent with the results of this study. It is worth noting that expression of heterologous genes in *E. coli* is often accompanied by the production of insoluble inclusion bodies. But inclusion body protein, in contrast, has its advantages—effective avoidance of degradation caused by endogenous protease and more favorable for purification by ultrasonic fragmentation. In this study, whilst the recombinant protein mainly expressed as inclusion body, its yield was relatively high and could be purified through nickel column and refolded into an active protein by dialysis (Figure 2).

After optimizing multiple reaction conditions by the checkerboard method (Figure 3), 100 clinical samples of sera were tested to plot ROC curves. The area under curve (AUC) within ROC showed a value of 0.932, reflecting a virtuous accuracy of rE2d-ELISA (Figure 4). Furthermore, based on the sensitivity of 0.82 and specificity of 0.90, the maximum Yorden index was calculated to be 0.72, and corresponding cut-off value was 0.396, which is similar to the threshold value (0.385) of the GETV antibody assay established by a previous report (Kuwata et al., 2018). The developed rE2d-ELISA method was used to detect five kinds of quine positive serums against widespread bacteria (tetanus and Salmonella) and viruses (EIV, EIAV, and RRV) in horses. In agreement with other studies (Bannai et al., 2019, 2020), only the cross-reactivity between rE2d and RRV positive serum was observed (Figure 5A), which may be due to the fact that GETV, together with other alphavirus such as RRV, chikungunya virus, and semliki forest virus, forms a serological complex group and therefore one of them has the characteristics of serological cross-reaction with each other according to the classification of antigenic relationships (Chen et al., 2018). Consequently, retesting with RRV-specific antibody detection methods is warranted if the sample originates from RRV-endemic areas as well as the OD_450_ is slightly greater than the cut-off value. When using ELISA for detection, generally the intra-batch CV should be less than 10% and the inter-batch CV should be less than 15% (Doherr et al., 1997). CVs of intra-batch and inter-batch of this method ranged from 3.5% ~ 8.3 to 6.3% ~ 8.1% respectively, both of which were within a reasonable parameter, suggesting that this ELISA method had good reproducibility. Since there is no commercialized GETV vaccine in China, the test results could exclude the effect of vaccine-induced immunity.

Given that lacking commercialized diagnostic kits for GETV antibodies and VNT, as one of the gold criteria for the serological diagnosis of GETV, is thought to be more specific in distinguishing GETV from other antigenically related alphaviruses (Fukunaga et al., 2000), many reported studies utilized VNT to carried out seroepidemiological investigation for GETV and to compare with their established detection methods(Li et al., 2019; Shi et al., 2022). In this study, 646 clinical serum samples collected from Xinjiang (Changji, Ili, Aksu and Kashi) were detected by rE2d-ELISA. Compared with VNT, the positive rate of IgG antibodies was 54.3% (351/646), slightly higher than that of neutralizing antibody 51.7% (334/646) whereas the total coincidence rate was 95.5% with a Kappa value of 0.879, indicating that the diagnostic results of these two approaches were in good consistency. The positive rate of these four regions ranged from 50.6 to 60.2% (Figure 6), but there was no significant difference among regions (*p* > 0.05). In comparison to other regions, the positive rate of GETV in horse in Xinjiang was higher than 25% in Hainan (Li et al., 1992), 24% in Hong Kong (Shortridge et al., 1994), and 17% in India (Brown and Timoney, 1998), but less than 93% in Japan after GETV became popular (Imagawa et al., 1981). Moreover, the GMT value of neutralizing antibody in 334 positive horse serum samples was calculated to be 1:135.8, combined with a high positive rate, speculating that GETV infection may be prevalent in horse populations in Xinjiang, which was similar to a previous study on the positive rate of GETV in Xinjiang from 2017 to 2020 (Shi et al., 2022).

The high positive rate in Xinjiang in this study may be influenced by the following factors. First, low setting of seropositive threshold. As there is no uniform standard for the division of seropositive threshold for detection of GETV antibodies by VNT, such as 1:4 (Bannai et al., 2019, 2020) and 1:10 (Kuwata et al., 2018; Li et al., 2019), in this study, a serum with neutralizing antibody titer ≥ 1:4 was defined as positive in order to calculate the GMT value (1:135.8) in Xinjiang as accurate as possible. Second, seasonal factor. Since the samples were collected from June to September 2021, the time of a whole year when mosquito activity was the most frequent, and the most important route of transmission of GETV is through mosquito bites (Lu et al., 2020), the number of horses infected by mosquito bites during this period may increase dramatically and so did the rate of positivity. From this perspective, this study only represents the prevalence of GETV disease in Xinjiang in the summer of 2021.

## Conclusion

An indirect ELISA method was established in the present study by using a recombinant protein E2d from GETV, which can facilitate the development of a reliable tool for the large-scale detection of GETV in horses. Meanwhile our study provides a basis for further understanding of the prevalence of GETV disease and an insight that constant monitoring for GETV in horses from Xinjiang, China should be strengthened.

## Data availability statement

The original contributions presented in the study are included in the article/Supplementary material, further inquiries can be directed to the corresponding authors.

## Author contributions

NJ and HL contributed to the conception of the study. XQ, XC, and NS designed the study and carried out the ELISAs. HZ, XZ, and YG analyzed and interpreted the data. ZM collected the serum samples. XQ and XC drafted the manuscript. All authors contributed to the article and approved the submitted version.

## Funding

This work was supported by the National Key Research and Development Program of China (2021YFC2301704), CAMS Innovation Fund for Medical Sciences (2020-12M-5-001) and National Natural Science Foundation of China project (32002322).

## Conflict of interest

The authors declare that the research was conducted in the absence of any commercial or financial relationships that could be construed as a potential conflict of interest.

## Publisher’s note

All claims expressed in this article are solely those of the authors and do not necessarily represent those of their affiliated organizations, or those of the publisher, the editors and the reviewers. Any product that may be evaluated in this article, or claim that may be made by its manufacturer, is not guaranteed or endorsed by the publisher.
