# Supplementary material for: Development and application of an indirect ELISA for detecting equine IgG antibodies against Getah virus with recombinant E2 domain protein
Source: Front Microbiol. 2022 Nov 10;13:1029444. doi: 10.3389/fmicb.2022.1029444 (PMC9685671; doi:10.3389/fmicb.2022.1029444)
Supplement: Supplementary file 1 [file Table_1.docx]

| **Supplement Table 1. E2 domian of nucleotide/amino acid homology analysis between SD17/09 and other GETV strains** | | | | | | | | | |
| --- | --- | --- | --- | --- | --- | --- | --- | --- | --- |
| **NO.** | **Genbank** | **Strain** | **Nucleotide**  **(%)** | **amino acid**  **(%)** | **NO.** | **Genbank** | **Strain** | **Nucleotide**  **(%)** | **amino acid**  **(%)** |
| **1** | AB032553.1 | GETV-SAGV | **96.8** | 97.3 | **36** | KY457548.1 | GZ0881 | 97.6 | 99.4 |
| **2** | AB859822.1 | Kochi/01/2005 | 99.4 | 99.7 | **37** | KY457549.1 | GZ0885 | 97.5 | 99.4 |
| **3** | AY702913.1 | South_Korea | 98 | 99.4 | **38** | KY457550.1 | GZ0896 | 97.6 | 99.4 |
| **4** | EF011023.1 | Alphavirus-M1 | 97.9 | 98.8 | **39** | KY457551.1 | GZ08133 | 97.5 | 99.4 |
| **5** | EF631998.1 | LEIV-16275-Mag | 97.5 | 99.1 | **40** | KY457552.1 | GZ08142 | 97.4 | 99.4 |
| **6** | EF631999.1 | LEIV-17741-MPR | 98.5 | 99.4 | **41** | LC079086.1 | MI-110-C1 | 98.4 | 99.1 |
| **7** | EU015061.1 | M1 | 97.8 | 98.5 | **42** | LC079087.1 | MI-110-C2 | 98.5 | 99.4 |
| **8** | EU015062.1 | HB0234 | 97.2 | 98.8 | **43** | LC079088.1 | 14-I-605-C1 | 97.5 | 99.4 |
| **9** | EU015063.1 | YN0540 | 97.6 | 99.4 | **44** | LC079089.1 | 14-I-605-C2 | 97.5 | 99.4 |
| **10** | EU015064.1 | YN0542 | 97.7 | 99.4 | **45** | LC107870.1 | SC1210 | 97.3 | 98.8 |
| **11** | EU015065.1 | HB0215-3 | 97.3 | 98.8 | **46** | LC152056.1 | 12IH26 | 97.6 | 99.4 |
| **12** | EU015066.1 | SH05-6 | 97.5 | 99.4 | **47** | LC212972.1 | 15-I-752 | 97.5 | 99.4 |
| **13** | EU015067.1 | SH05-15 | 97.6 | 99.4 | **48** | LC212973.1 | 15-I-1105 | 97.5 | 99.4 |
| **14** | EU015068.1 | SH05-16 | 97.6 | 99.4 | **49** | LC223130.1 | 16-I-599 | 97.5 | 99.4 |
| **15** | EU015069.1 | SH05-17 | 97.4 | 99.1 | **50** | LC223131.1 | 16-I-674 | 97.5 | 99.4 |
| **16** | EU015070.1 | GS10-2 | 97.6 | 99.1 | **51** | LC223132.1 | 16-I-676 | 97.5 | 99.4 |
| **17** | KP216576.1 | 12-YJ020 | 97.4 | 99.4 | **52** | LC534253.1 | SW | 95.2 | **97** |
| **18** | KR081238.1 | QIAG9301 | 98 | 99.4 | **53** | MF741771.1 | HuN1 | 99.7 | **100** |
| **19** | KR081239.1 | QIAG9302 | 98.3 | 99.1 | **54** | MG865965.1 | AH9192 | 97.4 | 98.8 |
| **20** | KR081240.1 | QIAG9303 | 98.2 | 98.8 | **55** | MG865966.1 | HNNY-1 | 97.6 | 99.4 |
| **21** | KY363862.1 | HNJZ-S1 | 97.4 | 99.4 | **56** | MG865967.1 | HNNY-2 | 97.4 | 99.4 |
| **22** | KY363863.1 | HNJZ-S2 | 97.6 | 99.4 | **57** | MG865968.1 | HNPDS-1 | 97.6 | 99.4 |
| **23** | KY399029.1 | GETV-V1 | 97.6 | 99.1 | **58** | MG865969.1 | HNPDS-2 | 97.6 | 99.4 |
| **24** | KY434327.1 | YN12031 | 96 | 97.3 | **59** | MG869691.1 | JL1708 | 97.5 | 99.1 |
| **25** | KY434328.1 | DY0824 | 97.7 | 99.4 | **60** | MH722255.1 | JL1707 | 97.2 | 98.5 |
| **26** | KY450683.1 | YN12042 | 97.6 | 99.4 | **61** | MH722256.1 | JL1808 | **99.9** | **100** |
| **27** | KY450684.1 | TC07180 | 97.7 | 99.4 | **62** | MK487997.1 | GZ201808 | 97.6 | 99.1 |
| **28** | KY450685.1 | LH07012 | 97.6 | 98.8 | **63** | MK693225.1 | SC201807 | 97.9 | 99.1 |
| **29** | KY450686.1 | DH10M1102 | 97.5 | 99.1 | **64** | MN478486.1 | SC483 | 97.8 | 98.8 |
| **30** | KY450687.1 | DH10M1106 | 97.6 | 99.4 | **65** | MN478487.1 | SC266 | 97.6 | 98.5 |
| **31** | KY450688.1 | DH10M390 | 97.6 | 99.4 | **66** | MN849355.1 | MM2021 | 94.4 | **97** |
| **32** | KY450689.1 | DH10M1105 | 97.6 | 99.4 | **67** | MT086508.1 | GDFS2-2018 | 97.6 | 99.1 |
| **33** | KY457545.1 | GZ0809 | 97.7 | 99.4 | **68** | MT086509.1 | GDFS9-2018 | 97.6 | 99.1 |
| **34** | KY457546.1 | GZ0862 | 97.5 | 99.4 | **69** | MT269657.1 | GX201808 | 99 | 99.7 |
| **35** | KY457547.1 | GZ0867 | 97.6 | 99.4 | **70** | NC_006558.1 | Getah_virus | 98 | 99.4 |
